# mTOR sustains inflammatory response in celiac disease

**DOI:** 10.1038/s41598-020-67889-4

**Published:** 2020-07-01

**Authors:** S. Sedda, V. Dinallo, I. Marafini, E. Franzè, O. A. Paoluzi, R. Izzo, P. Giuffrida, A. Di Sabatino, G. R. Corazza, G. Monteleone

**Affiliations:** 1 Department of Systems Medicine, University of “Tor Vergata”, Via Montpellier, 1, 00133 Rome, Italy; 2 Medpace Spain, Madrid, Spain; 3 Dipartimento Di Medicina Interna, Fondazione IRCCS Policlinico San Matteo, Università Di Pavia, Pavia, Italy

**Keywords:** Interleukins, Coeliac disease

## Abstract

Celiac disease (CD) is an enteropathy triggered by the ingestion of gluten proteins in genetically predisposed individuals and characterized by excessive activation of effector immune cells and enhanced production of inflammatory cytokines. However, factors/mechanisms that amplify the ongoing mucosal inflammation in CD are not fully understood. In this study, we assessed whether mammalian target of Rapamycin (mTOR), a pathway that combines intra- and extra-cellular signals and acts as a central regulator for the metabolism, growth, and function of immune and non-immune cells, sustains CD-associated immune response. Our findings indicate that expression of phosphorylated (p)/active form of mTOR is increased in protein lysates of duodenal biopsy samples taken from patients with active CD (ACD) as compared to normal controls. In ACD, activation of mTOR occurs mainly in the epithelial compartment and associates with enhanced expression of p-4EBP, a downstream target of mTOR complex (mTORC)1, while expression of p-Rictor, a component of mTORC2, is not increased. Stimulation of mucosal explants of inactive CD patients with pepsin-trypsin-digested (PT)-gliadin or IFN-γ/IL-21, two cytokines produced in CD by gluten-specific T cells, increases p-4EBP expression. Consistently, blockade of such cytokines in cultures of ACD mucosal explants reduces p-4EBP. Finally, we show that inhibition of mTORC1 with rapamycin in ACD mucosal explants reduces p-4EBP and production of IL-15, a master cytokine produced by epithelial cells in this disorder. Our data suggest that ACD inflammation is marked by activation of mTORC1 in the epithelial compartment.

## Introduction

Celiac disease (CD) is an immune-mediated disease, which arises in genetically predisposed individuals, followed ingestion of gluten proteins^1^. Histologically, CD is marked by various degrees of villous atrophy, crypt hyperplasia and enhanced T lymphocyte infiltration in the epithelial compartment and lamina propria. In CD patients, ingestion of gluten stimulates innate and adaptive immune cells to produce various cytokines, which contribute to the CD-associated pathological process^2–5^. In particular, gluten peptides stimulate both epithelial cells and innate immune cells to secrete interleukin (IL)-15^6^, which in turn activates both CD8+ and natural killer + cells thereby contributing to the gluten-driven epithelial damage^1,7^. Moreover, gluten exposure facilitates differentiation of CD4+ T-cells in T helper (Th)-type 1 cells^8,9^. These gluten-specific Th1 lymphocytes synthesize interleukin (IL)-21 and interferon (IFN)-γ, two cytokines that activate several intracellular pathways and contribute to establish positive feedback loops amplifying the ongoing mucosal immune response^10–12^. For instance, IFN-γ triggers activation of signal transducer and activator of transcription (STAT)-1 and induces of T-bet, a transcription factor essential for IFN-γ production^13–16^. Additionally, IL-21 enhances expression of T-be and IFN-γ in CD4+ Th1 cells as well as stimulates fibroblasts to secrete extracellular matrix metalloproteinases^12,17,18^. Since both IL-21 and IFN-γ can also target antigen presenting cells and epithelial cells^13,19–21^, it is conceivable that these two cytokines contribute to expand innate immune responses in CD.

The mammalian target of Rapamycin (mTOR) is a serine/threonine kinase belonging to the phosphatidylinositol 3-Kinase (PI3K)-related kinase superfamily, which regulates the metabolism of cells according to their need to both differentiate and proliferate^22,23^. mTOR responds to extracellular signals, such as growth factors and hormones, ligation of pattern recognition and antigen-specific receptors (e.g. B-cell receptor, T-cell receptor, toll like receptors), cytokines (e.g. IL-12, IL-4, IL-2,) and intracellular signals including nutrients, and cellular energy charge^24–26^. Active phosphorylated (p)-mTOR forms two distinct multiprotein complexes: mTOR complex 1 (mTORC1) and mTORC2. mTORC1 consists of 5 protein components [mammalian lethal with Sec13 protein 8 (mLST8), mTOR, regulatory protein associated with mTOR (Raptor), DEP domain containing mTOR interacting protein (Deptor), and proline-rich Akt substrate of 40 kDa (PRAS40)] and regulates the activation (phosphorylation) of downstream targets, such as p70S6 Kinase 1 (S6K1) and eIF4E Binding Protein (4EBP), thus leading to cell growth and protein synthesis^27,28^. mTORC2 consists of 6 protein components [mammalian stress-activated protein-interacting protein 1 (mSIN1), mLST8, mTOR, rapamycin insensitive companion of mTOR (Rictor), protein observed with Rictor-1 (Protor-1), and Deptor]^28^. The role of mTORC2 is not fully understood, even though it has been shown that mTORC2 regulates actin organization and phosphorylates protein kinase B (Akt) at serine 473, thus exerting a positive feedback in the cascade of mTOR itself^23^. The critical role of mTOR pathway in cellular proliferation/survival has been substantiated by the presence of constitutively activated or mutated forms of mTOR and its upstream and downstream signaling proteins in different malignant disorders^29^. mTOR kinase cascade can also control T cell differentiation, as it is capable of facilitating polarization of both Th1 and Th17 cells and inhibiting the differentiation of regulatory T cells^30^. Moreover, excessive activation of mTOR promotes differentiation of classical, type I macrophages, a subset of macrophages that synthesize inflammatory cytokines^31^. These findings well fit with the demonstration that hyper-activation of mTOR occurs in many immune-inflammatory diseases^32,33^. The aim of this study was to investigate the role of mTOR kinase cascade in the CD-related mucosal inflammation.

## Results

### p-mTOR is upregulated in the duodenum of active celiac disease patients

**Figure 1:**
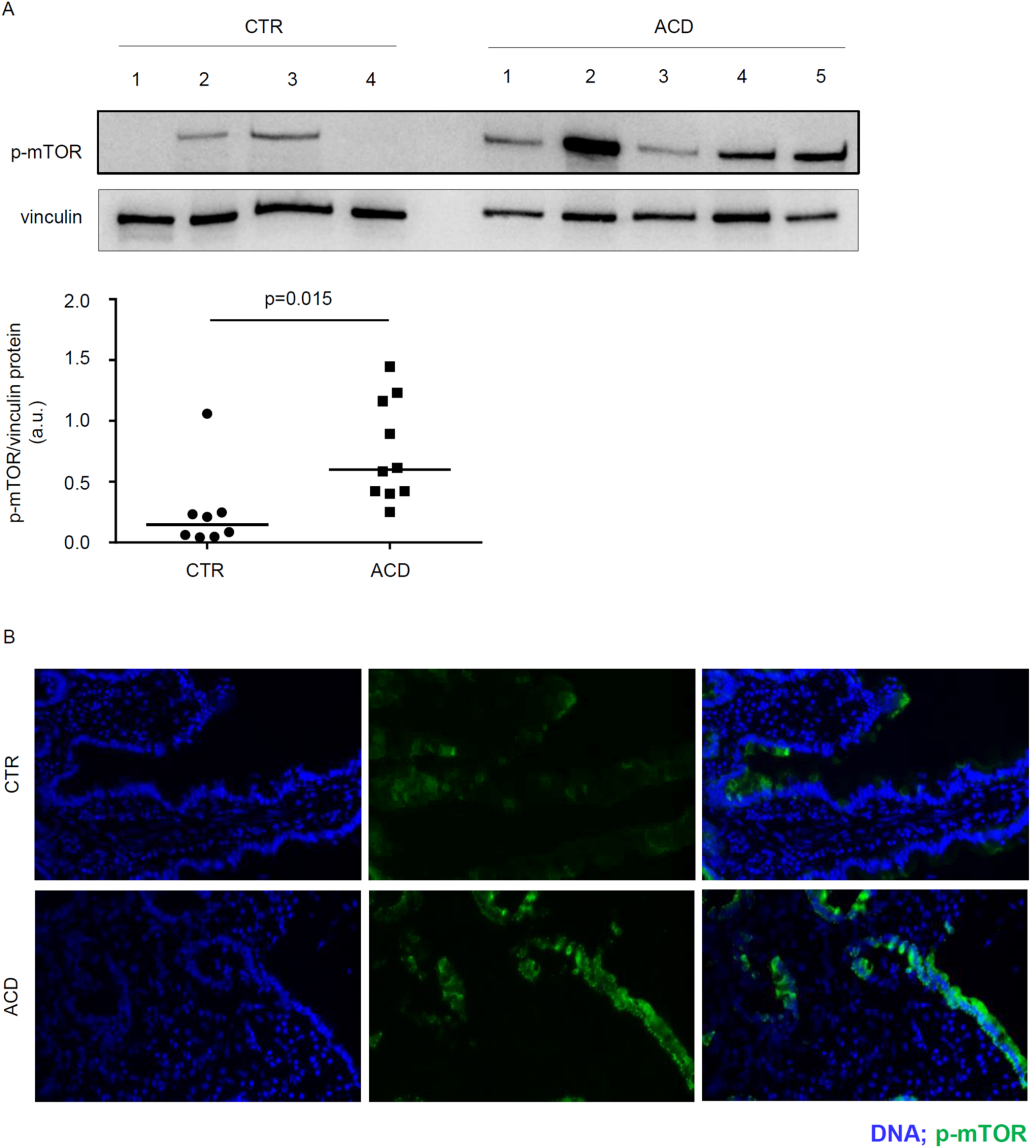
Phosphorylated (p)/active form of the mammalian target of Rapamycin (mTOR) is up-regulated in active celiac disease patients. (**A**) Western blots showing p-mTOR and vinculin (used as a loading control) expression in duodenal biopsy samples taken from 4 normal controls (CTR) and 5 active celiac disease (ACD) patients. The blots are representative of 2 separate experiments in which total proteins extracted from duodenal biopsy samples of 8 CTR and 10 ACD were analyzed. The panel shows quantitative analysis of p-mTOR/vinculin ratio in each sample as measured by densitometry scanning of all Western blots; horizontal bar indicates median value. Values are expressed in arbitrary units (a.u.). (**B**) Representative images of immunofluorescence staining of duodenal sections of CTR and ACD patients for p-mTOR (green staining). DNA is stained with DAPI (blue).

To determine whether mTOR pathway is active in CD, total proteins extracted from biopsy samples of patients with active CD (ACD) and normal controls were analysed for the phosphorylated/active form of mTOR by Western blotting. p-mTOR expression was more pronounced in ACD as compared to controls (Fig. 1A). Densitometric analysis of immunoblots confirmed that p-mTOR expression was significantly higher in ACD than in controls (Fig. 1A, lower panel).

To assess which cells express p-mTOR in the duodenum, we performed immunofluorescence analysis using frozen sections of ACD and control duodenal biopsy samples. A constitutive but very faint staining for p-mTOR was evident in the epithelium of normal controls (Fig. 1B). p-mTOR expression was more pronounced in the epithelial cells of ACD patients as compared to controls (Fig. 1B). In both normal controls and ACD patients, p-mTOR staining was virtually absent in the lamina propria compartment.

### mTORC1 pathway is activated in CD

**Figure 2:**
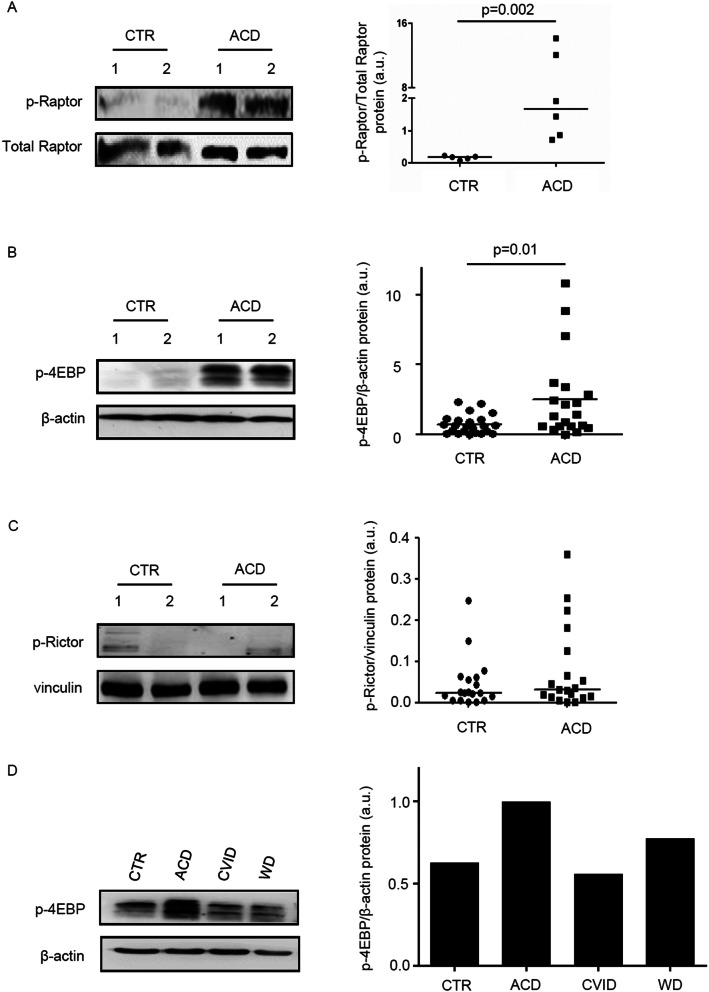
mTOR complex 1 (mTORC1) is activated in active celiac disease. (**A**) Western blots showing phosphorylated (p) and total forms of Raptor in duodenal biopsy samples taken from 2 normal controls (CTR) and 2 active celiac disease (ACD) patients. The blots are representative of 2 separate experiments in which total proteins extracted from duodenal biopsy samples of 5 CTR and 6 ACD patients were analyzed. The right panel shows quantitative analysis of p-Raptor/total Raptor ratio in each sample as measured by densitometry scanning of all Western blots; horizontal bar indicates median value. Values are expressed in arbitrary units (a.u.). (**B**) Western blots showing phosphorylated (p)-4E-binding protein (4EBP) and β-actin (used as a loading control) expression in duodenal biopsy samples taken from 2 normal controls (CTR) and 2 active celiac disease (ACD) patients. The blots are representative of 6 separate experiments in which total proteins extracted from duodenal biopsy samples of 23 CTR and 20 ACD were analyzed. The right panel shows quantitative analysis of p-4EBP/β-actin ratio in each sample as measured by densitometry scanning of all Western blots; horizontal bar indicates median value. Values are expressed in arbitrary units (a.u.). (**C**) Western blots showing phosphorylated (p)-Rapamycin-insensitive companion of mammalian target of rapamycin (Rictor) and vinculin (used as a loading control) expression in duodenal biopsy samples taken from 2 CTR and 2 ACD patients. The blots are representative of 2 separate experiments in which total proteins extracted from duodenal biopsies of 19 CTR and 19 ACD were analyzed. The right panel shows quantitative analysis of p-Rictor/vinculin ratio in each sample as measured by densitometry scanning of all Western blots; horizontal bar indicates median value. Values are expressed in arbitrary units (a.u.). (**D**) Western blots showing p-4EBP and β-actin (used as a loading control) expression in duodenal biopsy samples taken from 1 CTR, 1 patient with ACD, 1 patient with Whipple disease (WD) and 1 patient with common variable immunodeficiency (CVID). The right panel shows quantitative analysis of p-4EBP/β-actin ratio as measured by densitometry scanning of all Western blots.

mTOR interacts with other proteins thus leading to the formation of two complexes, namely mTORC1 and mTORC2^28^. To determine which complex is activated in CD mucosa, we initially evaluated expression of p-Raptor, a component of the mTORC1 complex^28,34^, in whole biopsy samples by Western blotting. Immunoreactive bands for p-Raptor were more evident in ACD as compared to controls (Fig. 2A). Next, we analysed expression of p-4EBP, an indicator of mTORC1 activation^28,34^, and p-Rictor, a component of mTORC2 complex^28,35^. p-4EBP was more expressed in ACD than in controls (Fig. 2B). Densitometric analysis of immunoblots confirmed the more pronounced expression of p-Raptor and p-4EBP in ACD as compared to controls (Fig. 2A,B, right panels). In contrast, p-Rictor expression did not differ between ACD patients and normal controls (Fig. 2C). To evaluate whether changes in mTOR kinase cascade are simply due to the ongoing duodenal inflammation, we analysed p-4EBP in duodenal samples of 2 patients with Whipple disease (WD) and 2 patients with common variable immunodeficiency (CVID). p-4EBP was detectable in all these samples with no apparent difference between CVID, WD, and controls (Fig. 2D).

### Activation of mTOR is induced by gliadin and inflammatory cytokines produced by gluten-specific T cell clones

**Figure 3:**
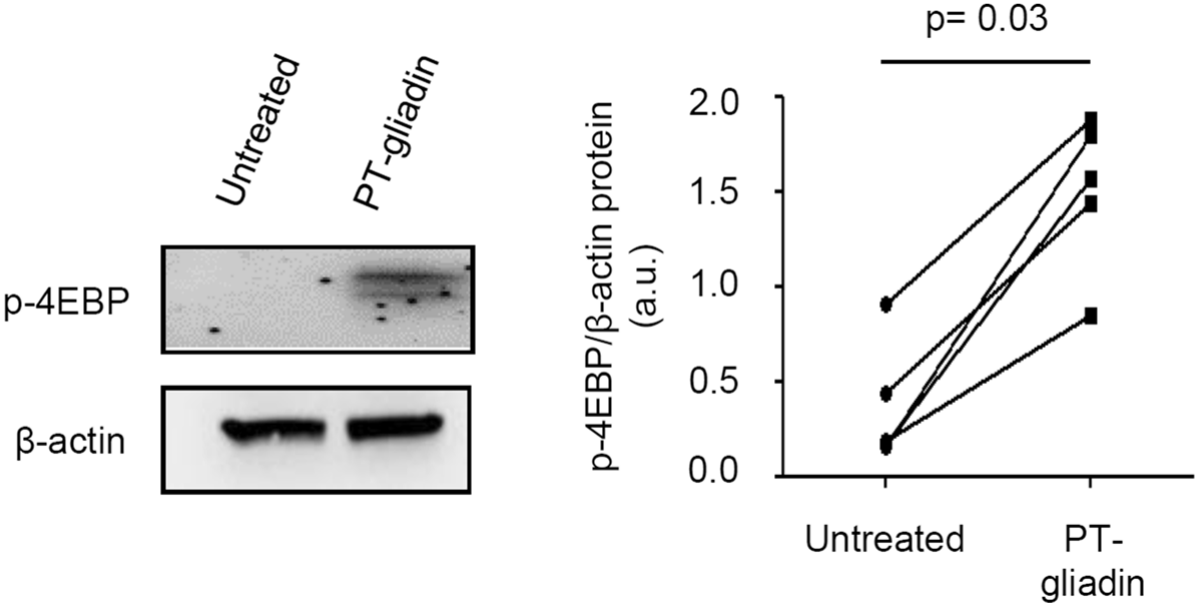
mTOR is induced by gluten-peptide in celiac disease. Duodenal biopsy samples taken from 5 inactive celiac disease (ICD) patients were cultured in the presence or absence of a pepsin-trypsin-digested (PT)-gliadin. After 24 h, total proteins were assessed for p-4EBP by Western blotting; β-actin was used as a loading control. The panel shows quantitative analysis of p-4EBP/β-actin ratio as measured by densitometry scanning of all Western blots. Values are expressed in arbitrary units (a.u.).

The fact that p-mTOR is more expressed in ACD than in controls prompted us to ascertain if this kinase is induced as a result of the gliadin-driven inflammatory response. To this end, duodenal biopsy samples of inactive celiac disease (ICD) patients, who were on a gluten-free diet (GFD), were cultured in the presence or absence of pepsin-trypsin-digested (PT)-gliadin and then assessed for p-4EBP expression by Western blotting. Stimulation of ICD samples with PT for 24 h consistently enhanced p-4EBP expression (Fig. 3).

**Figure 4:**
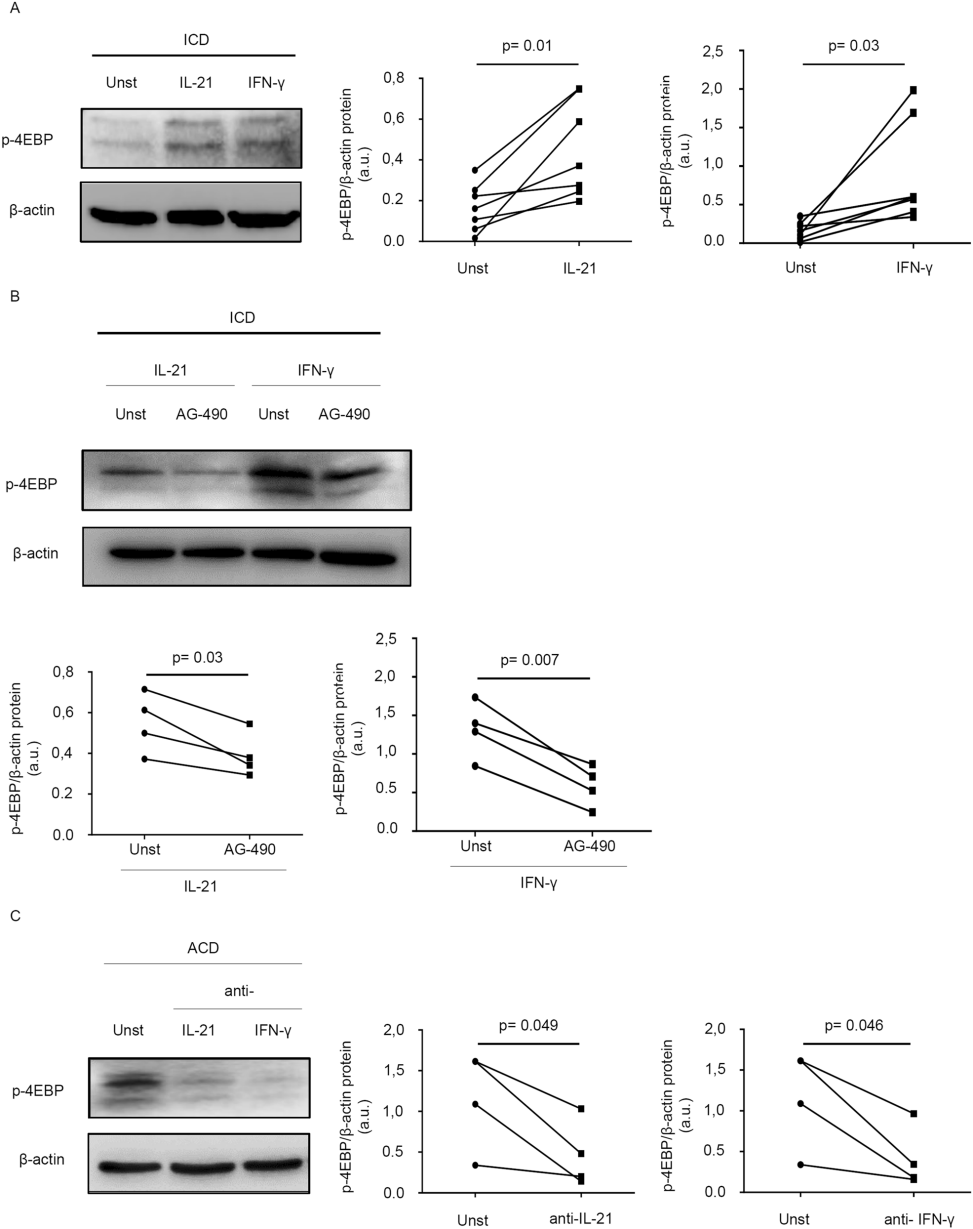
Interleukin (IL)-21 and interferon (IFN)-γ induce mTOR activation in celiac disease. (**A**) Duodenal biopsy samples taken from inactive celiac disease (ICD) patients were either left unstimulated (Unst) or stimulated with IL-21 or IFN-γ. After 24 h, total proteins were assessed for p-4EBP by Western blotting; β-actin was used as a loading control. The blots are representative of 7 separate experiments in which similar results were obtained. The right insets show quantitative analysis of p-4EBP/β-actin ratio as measured by densitometry scanning of all Western blots. Values are expressed in arbitrary units (a.u.). (**B**) Duodenal biopsy samples taken from 4 ICD patients were either left untreated (Unt) or treated with the Janus kinase/signal transducers and activators of transcription (JAK/STAT) inhibitor AG490 in the presence or absence of IL-21 or IFN-γ. After 24 h, total proteins were assessed for p-4EBP by Western blotting; β-actin was used as a loading control. The blots are representative of 4 separate experiments in which similar results were obtained. The insets show quantitative analysis of p-4EBP/β-actin ratio as measured by densitometry scanning of all Western blots. Values are expressed in arbitrary units (a.u.). (**C**) Duodenal biopsy samples taken from 4 active celiac disease (ACD) patients were either left untreated (Unt) or treated with antibodies neutralizing IL-21 or IFN-γ, After 24 h, total proteins were assessed for p-4EBP by Western blotting; β-actin was used as a loading control. The blots are representative of 4 separate experiments in which similar results were obtained. The right insets show quantitative analysis of p-4EBP/β-actin ratio as measured by densitometry scanning of all Western blots. Values are expressed in arbitrary units (a.u.).

These findings suggest that activation of mTOR occurs as an early event in the gliadin-induced immune response in ACD. CD-associated inflammation is marked by activation of gluten-specific T cell clones, which produce IFN-γ and IL-21, two cytokines that target epithelial cells in the gut^19,20,36^. Therefore, we next investigated the involvement of these 2 cytokines in the activation of mTOR. p-4EBP expression was evaluated in duodenal samples of ICD patients stimulated with IFN-γ or IL-21 by Western blotting. Both IFN-γ and IL-21 enhanced p-4EBP expression (Fig. 4A). Many biological functions of IFN-γ and IL-21 are mediated by the Janus kinase/signal transducers and activators of transcription (JAK/STAT) pathway^16,37^. Therefore, ICD duodenal samples were stimulated with such cytokines in the presence or absence of a JAK/STAT inhibitor (AG-490). In all the experiments, treatment of biopsy samples with AG490 reduced IFN-γ/IL-21-induced p-4EBP expression (Fig. 4B). To confirm the inducing effect of IFN-γ and IL-21 on mTOR activation, p-4EBP expression was next evaluated in duodenal samples of ACD patients treated with antibodies neutralizing IFN-γ or IL-21. As shown in Fig. 4C, neutralization of either IFN-γ or IL-21 reduced p-4EBP expression.

### Inhibition of mTOR reduces IL-15 expression

**Figure 5:**
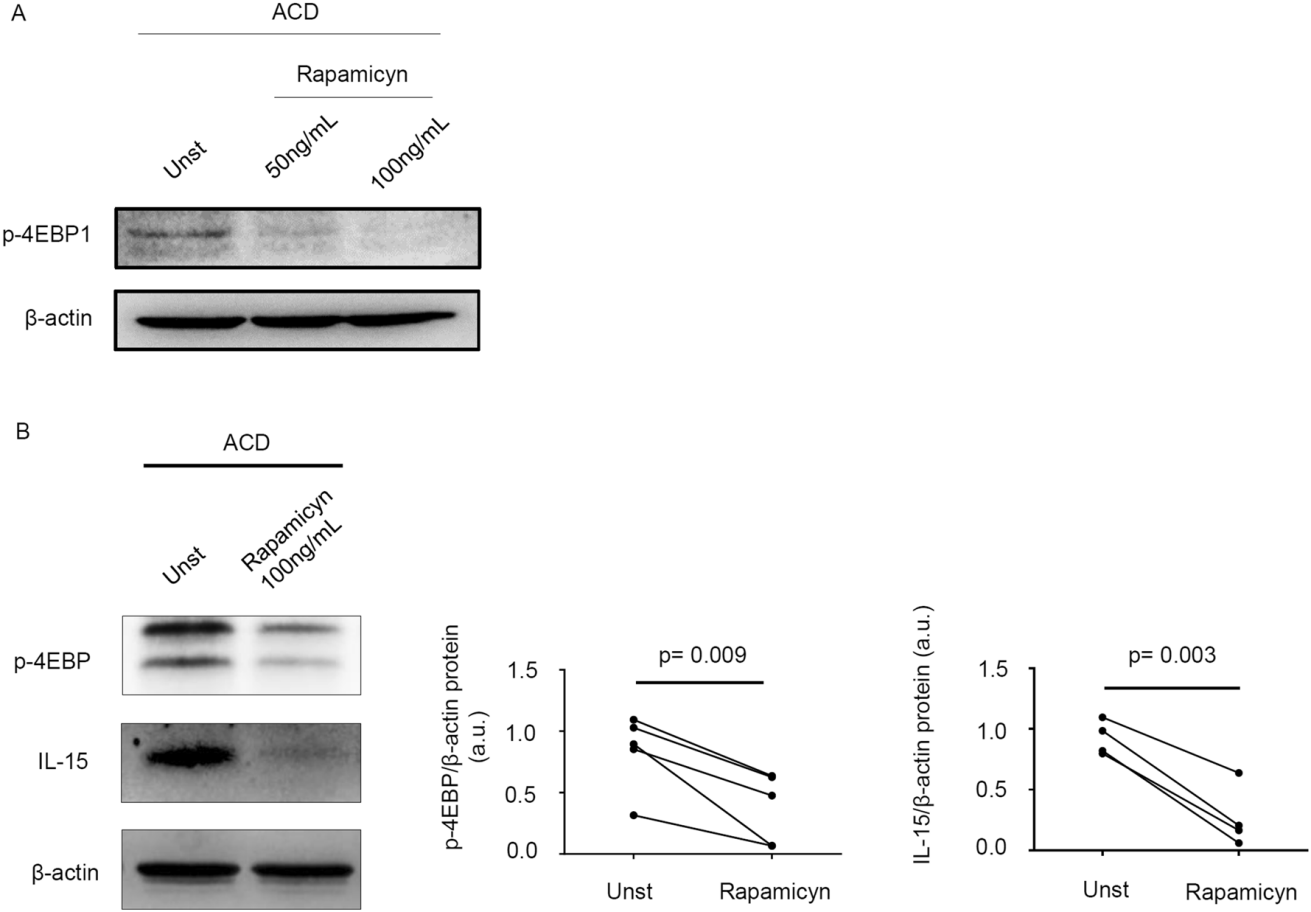
Inhibition of mTOR signalling reduces interleukin (IL)-15 expression in active celiac disease. (**A**) Representative Western blot showing p-4EBP expression in duodenal biopsy samples taken from 1 patient with active celiac disease (ACD) either left untreated (Unt) or treated with 50 ng/mL, 100 ng/mL rapamycin for 24 h; β-actin was used as a loading control. (**B**) Duodenal biopsy samples taken from 4 ACD patients were either left untreated (Unt) or treated with 100 ng/mL rapamycin for 24 h and p-4EBP and IL-15 expression was assessed by Western blotting. β-actin was used as a loading control. The blots are representative of 4 separate experiments in which similar results were obtained. The right insets show quantitative analysis of p-4EBP/β-actin and IL-15/β-actin ratio as measured by densitometry scanning of all Western blots. Values are expressed in arbitrary units (a.u.).

In a final set of experiments, we assessed whether mTOR regulates IL-15 synthesis, as this cytokine is produced by epithelial cells in CD^38^. For this purpose, duodenal samples of ACD patients were cultured in the presence or absence of rapamycin, an inhibitor of mTORC1, for 24 h and IL-15 protein expression was then evaluated by Western blotting. Rapamycin markedly inhibited p-4EBP expression (Fig. 5A,B) and this associated with a reduction of IL-15 production (Fig. 5B).

## Discussion

This study was undertaken to examine whether CD-associated inflammation is marked by change in the activation of mTOR, a signaling pathway that controls and coordinates the function of various immune cells and has been involved in the amplification of inflammatory signals in many immune-mediated disorders^22,33,34,39,40^. By using specific antibodies that recognize the phosphorylated, active form of mTOR, we initially showed that mTOR is constitutively activated in the human duodenum and expression of the active protein is markedly increased in ACD as compared to controls. Immunofluorescence analysis showed that epithelial cells were the only source of mTOR in both controls and patients and confirmed the more pronounced expression in ACD. These data are consistent with results of a recent study documenting hyper-activation of mTORC1 in the colonic epithelium of ulcerative colitis (UC) patients and in the epithelium of mice with dextran sulfate sodium-induced colitis^33^. mTOR functions depend on two distinct multiprotein complexes, namely mTORC1 and mTORC2^28^. mTORC1 is mostly involved in the regulation of the translation initiation machinery influencing cell growth, proliferation, and survival, while mTORC2 participates in actin cytoskeleton rearrangements and cell survival^28^. Data of the present study suggest that mTORC1 and not mTORC2 is hyper-activated in ACD mucosa. Indeed, we showed enhanced expression of p-Raptor, a component of mTORC1, and p-4EBP, a downstream effector of mTORC1 signaling, in duodenal samples of ACD as compared to normal controls while expression of p-Rictor, an indicator of mTORC2 complex activation, did not differ between ACD and controls. This is not surprising as a different expression of these 2 complexes has been described in type 2 diabetes, where mTORC1 hyper-activation in pancreatic islets associates with a decline in the expression of active mTORC2^34,35,41^. The exact factors/mechanisms that activate mTOR in ACD remain to be ascertain. It is likely that the sustained activation of this kinase is not simply due to the ongoing mucosal inflammation, as we were not able to detect any change in p-4EBP in the duodenum of patients with other enteropathies (i.e. CVID and WD). At the same time, our findings support the hypothesis that mTOR can be positively regulated by gluten-driven inflammation. Indeed, ex vivo treatment of ICD samples with PT enhanced p-4EBP. We do not yet know which sequence of events is needed to induce mTOR in PT-treated biopsy samples. A possibility is that PT actives directly mTOR in line with the demonstration that intestinal epithelial cells can respond to PT by increasing production of IL-15^42^. It is also conceivable that PT can trigger an immune response that ultimately controls mTOR activation. Support to this hypothesis comes from the demonstration that both IFN-γ and IL-21, two cytokines over-produced by gluten-specific Th1 cells in ACD^10,12^, were able to enhance p-4EBP in ICD duodenal samples while blockade of these two molecules with neutralising antibodies reduced p-4EBP in cultures of ACD biopsy samples.

The demonstration that PT enhances p-4EBP in ICD duodenal samples would seem to conflict with data of a recent study showing an inhibitory effect of gluten peptides on the activity of mTORC1 in blood monocytes isolated from CD patients. In particular, gluten digests, through the inhibition of mTOR, induced arginase 1 and 2 thus promoting differentiation of type2-like macrophages^43^. These divergent results reflect likely the different cell systems used to analyse mTOR function in response to PT stimulation. However, in this context, we would like to point-out that in the duodenum of CD patients activation of mTOR occurred mainly in the epithelial compartment rather than in myeloid cells.

Our study supports data of previous studies documenting hyperactivation of mTOR in other immune-mediated disorders. For instance, enhanced activation of mTOR has been seen in lesional psoriatic skin and there is evidence that Th1/Th17 inflammatory cytokines produced within the psoriatic lesion activate mTORC1 thus contributing to the excessive proliferation of the keratinocytes^39^. As pointed out above, mTORC1 is activated in inflamed colon of UC patients, where it is supposed to promote recruitment of Th17 cell in the colon via a STAT3/cyclooxygenase 2-dependent mechanism^33^. mTORC1-mediated Stat3 activation in intestinal epithelial cells can also stimulate the expression of downstream targets essential for cell proliferation and tissue regeneration, thus highlighting a protective role of mTORC1 in physiological conditions^44^. Activation of mTORC1 but not mTORC2 has been also described in patients with systemic lupus erythematosus (SLE) as well as in mice with SLE-like pathology^40^.

Production of IL-15 by epithelial cells and innate immune cells marks the CD-associated immune response and accumulating evidence suggests that this phenomenon is crucial in the pathogenesis of the disorder as IL-15 inhibits intraepithelial lymphocyte (IEL) apoptosis, induces IEL to proliferate and release proinflammatory cytokines^45^ and promotes perforin and granzyme-mediated cytotoxicity^1,7,38^. Here, we show that activation of mTOR of ACD patients contributes to sustain IL-15 production since inhibition of mTORC1 with rapamycin in cultures of ACD mucosal samples reduced IL-15 synthesis. These studies were carried-out using whole biopsy samples rather than primary epithelial cells because it is well-known that such cells die very quickly after isolation from the intestinal mucosa. Therefore, we cannot exclude the possibility that down-regulation of IL-15 in rapamicyn-treated duodenal samples can, in part, rely on the control of additional pathways in immune cells other than inhibition of mTORC1 in epithelial cells. We do not yet know the exact mechanism by which mTORC1 regulates IL-15. A possibility is that mTOR regulates positively nuclear factor kappa B (NF-kB)^46^, a transcription factor that stimulates IL-15 production^47^. mTOR could also regulate post-transcriptionally IL-15 production, as it is well known that IL-15 synthesis is also controlled at the translational level^48,49^ and mTOR signaling stimulates the initiation of translation in eukaryotic cells^50^.

We are aware that our study has some limitations. Some components of the mTOR cascade were analyzed in a limited number of patients due to the difficulty to collect simultaneously many biopsy samples during endoscopy. Therefore, further work is needed to confirm the present results. It remains unclear if there is a cell-specific regulation of mTOR activation in CD as the functional studies were performed using whole mucosal samples rather than single cell types. Further longitudinal studies will be also needed to ascertain the impact of gluten-free diet on mTOR activation. Although no mTOR-positive cell was localized in the lamina propria compartment of CD patients by immunohistochemistry, more sensitive techniques (e.g. flow-cytometry) could help establish if activation of such a kinase occurs in specific immune cell types.

In conclusion, our data suggest that ACD inflammation is marked by activation of mTORC1 in the epithelial compartment.

## Materials and methods

### Patients and samples

We enrolled 69 consecutive patients undergoing upper endoscopy at the Gastrointestinal Unit of Tor Vergata University Hospital (Rome, Italy) or San Matteo Hospital, University of Pavia (Pavia, Italy). The study population included: 29 ACD patients on a gluten-containing diet who were positive for both serum anti-endomysium IgA antibody (EMA) and anti-tissue transglutaminase 2 (TG-2) IgA antibody, evidenced respectively through indirect immunofluorescence and chemiluminescent immunoassay of serum samples, and had villous atrophy on histological examination according to Marsh classification (grade 3 A–C); 13 asymptomatic ICD patients, who were on a strict GFD, negative for both EMA and anti-TG-2 antibodies, and had normal duodenal histology. Controls included 27 dyspeptic patients, who were negative for both EMA and anti-TG-2 antibody while receiving a gluten-containing diet and had neither macroscopic nor histologic lesions (normal controls), 2 patients with WD and 2 patients with CVID (disease controls). Both CD patients and controls were free to use fermentable oligosaccharides disaccharides monosaccharides and polyols-containing products. Evaluation of the daily value of gluten exposure was not routinely made in our ACD population. However, patients reported to eat regularly bread, pasta and other gluten-containing foods, thus assuming their diet consisted of more than 5 g of gluten per day. Both the patients and controls had experienced no symptoms/signs suggestive for allergy to wheat (e.g. asthma, itching, hives, or anaphylaxis). After a strict gluten-free diet, all the 25 ACD patients were negative for both EMA and anti-TG2 and histological examination of duodenal biopsy samples taken from these patients showed no CD-associated mucosal change (Marsh classification; grade 0); Biopsy samples were taken from the duodenum and stored for protein extraction or embedded in a cryostat mounting medium and stored for immunofluorescence analysis. Each patient who took part in the study gave written informed consent. All methods were carried out in accordance with relevant guidelines and regulations. All experimental protocols were approved by the Independent Ethic Committee at the Policlinico Tor Vergata of Rome (Rome, Italy).

### Total protein extraction and western blotting

All reagents were from Sigma-Aldrich (Milan, Italy), unless specified. Biopsy samples were lysed on ice with a buffer containing 10 mM HEPES (ph 7.9), 0.1 mM EDTA, 10 mM KCl, and 0.5% Nonidet p40, supplemented with 1 mM dithiothreitol, 10 mg/ml leupeptin,1 0 mg/ml aprotinin, 1 mM Na3VO4, 1 mM phenyl-methylsulfonyl fluoride, and 1 mM NaF. Lysates were clarified by centrifugation at 12,000*g* for 30 min at 4 °C and separated on 15% sodium dodecyl sulphate (SDS)-polyacrylamide gel electrophoresis (for p-4EBP and IL-15) or on 6% SDS–polyacrylamide gel electrophoresis (for p-mTOR, p-Rictor and p-Raptor). p-4EBP, p-mTOR, p-Rictor and p-Raptor were detected using monoclonal rabbit anti-human antibodies (1:500 final dilution; Cell signalling technology, Leiden, The Netherlands) followed by a horseradish peroxidase–conjugated mouse anti-rabbit IgG monoclonal antibody (1:20,000 final dilution; Dako, Milan, Italy). IL-15 was detected using a monoclonal mouse anti-human antibody (1:500 final dilution; GeneTex, Irvine, CA), followed by a horseradish peroxidase–conjugated rabbit anti-mouse IgG monoclonal antibody (1:20,000 final dilution; Dako). The reactions were detected with a sensitive enhanced chemiluminescence kit (Pierce, Rockford, IL). After the analysis, blots were stripped and incubated with a mouse anti-human β-Actin antibody or a rabbit anti-human vinculin antibody, (1:5,000 final dilution; Abcam, Cambridge, MA) depending on the molecular size of the analysed protein, or a rabbit anti-human Raptor antibody (1: 500 final dilution, Cell signalling technology) followed by specific secondary antibodies conjugated to horseradish peroxidase, to ascertain equivalent loading of lanes.

### Immunofluorescence

Immunofluorescence was performed on frozen duodenal sections. Samples were embedded in a cryostat mounting medium (Neg–50 Frozen Section Medium, Thermo Scientific, Langenselbold, Germany), snap frozen and stored at − 80 °C. Six μm-thickened sections were mounted onto superfrost plus glass slides (Thermo Scientific) and fixed in 4% paraformaldehyde (PFA) for 10 min at 4 °C. Slides were washed three times with TBS, treated with 0.1% Triton X-100 for 20 min at room temperature (RT). Blocking was performed with a 10% normal goat serum TBS solution for 1 h at RT. Slides were then incubated overnight (ON) at 4 °C with a monoclonal rabbit anti-human p-mTOR antibody (1:50 final dilution, Cell signalling technology). After washing three times with TBS, slides were incubated for 1 h at RT with a specific secondary antibody coupled with Alexa Fluor Dye (1:2,000 final dilution; Invitrogen, Milan, Italy). Coverslips were mounted on glass slides using ProLong Gold antifade reagent with DAPI (Invitrogen) to counterstain the DNA. Images were acquired on a Leica DMI 4,000 B fluorescence microscope (Leica, Wetzlar, Germany).

### Organ cultures

Mucosal biopsy samples of ICD patients and normal controls were placed on transwell (transwell permeable support, Costar, Corning incorporated, New York, USA) in 24-well plates containing RPMI medium supplemented with 1% P/S and 50 μg/ml gentamycin in the absence or presence of PT (1 mg/ml). In addition, mucosal biopsy samples of ICD patients were placed on transwell in 24-well plates containing RPMI medium supplemented with 1% P/S and 50 μg/ml gentamycin in the presence of IFN-γ or IL-21 (50 ng/ml final concentration, Peprotech, London, UK). In parallel, samples were cultured with or without cytokines in the presence or absence of the JAK/STAT inhibitor AG-490 (100 μM final concentration, Inalco, Milan, Italy). Mucosal biopsy samples of ACD patients were cultured as above in the presence of antibodies neutralizing IFN-γ or IL-21 (both used at 10 μg/ml final concentration, R&D Systems, Minneapolis, MN, USA). In further experiments, mucosal biopsy samples of ACD patients were cultured in the presence or absence of rapamycin (50 or 100 ng/ml final concentration).

The cultures were performed in an organ culture chamber at 37 °C in a 5% CO_2_/95% O_2_ atmosphere and after 24 h the samples were used for protein extraction.

### Data analysis

Differences between groups were compared using the two-tailed T-test and Mann–Whitney U test. Statistical differences were assessed with the GraphPad Prism statistical PC program (GraphPad Software, San Diego, CA). A p value of less than 0.05 was considered statistically significant.
